# Behavioral and Sociodemographic Determinants of Hypertension and Its Burden among Bank Employees in Metropolitan Cities of Amhara Regional State, Ethiopia

**DOI:** 10.1155/2021/6616473

**Published:** 2021-07-16

**Authors:** Kegnie Shitu, Ayenew Kassie

**Affiliations:** Department of Health Education and Behavioral Sciences, Institute of Public Health, College of Medicine and Health Sciences, University of Gondar, Gondar, Ethiopia

## Abstract

**Background:**

Hypertension is the leading cause of cardiovascular disease and premature death worldwide. Bank workers are at higher risk of hypertension because of their work sedentary characteristics. However, little is known about the prevalence and determinants of hypertension among this group of population. Therefore, this study aimed to assess the prevalence and associated factors of hypertension among bank employees in metropolitan cities in Amhara Regional State of Ethiopia.

**Method:**

An institution-based cross-sectional study was conducted among 368 bank employees. A simple random sampling technique was used to select participants. A pretested self-administered questionnaire and biophysical measurements were employed to collect the data. Descriptive statistics and logistic regression analyses were done to summarize the data and identify factors associated with hypertension, respectively.

**Result:**

The overall prevalence of hypertension among bank employees was 52.4% (95% CI: 47.2, 57.7). Increased age (AOR = 1.1, 95% CI: 1.03, 1.11), male sex (AOR = 2.5, 95% CI: 1.2, 5.1), overweight (AOR = 2.7, 95% CI: 1.5, 5.2), obesity (AOR = 5.6, 95% CI: 2.0, 11.3), moderate/high physical activity (AOR = 0.36, 95% CI: 0.2, 0.62), daily fruit intake (AOR = 0.1, 95% CI: 0.04, 0.3), stressful life event experience (AOR = 1.8, 95% CI: 1.01, 3.4), family history of hypertension (AOR = 2.8, 95% CI: 1.5, 5.4), and poor knowledge of CVDs (AOR = 2.4, 95% CI: 1.2, 4.8) were significantly associated with hypertension.

**Conclusion:**

The prevalence of hypertension among bank workers was very high. Increased age, male sex, overweight and obesity, daily fruit intake, moderate/high physical activity, the experience of stressful events, familial history of hypertension, and poor CVDs knowledge were associated with hypertension. Thus, raising awareness about cardiovascular disorders and behavior change interventions that enhance bank workers' engagement in physical exercise, screening behavior, and a healthy diet is urgently required for this group of population.

## 1. Introduction

Hypertension is also known as high or raised blood pressure (BP). About 1.13 billion people worldwide have hypertension, most (two-thirds) living in low- and middle-income countries [1]. This figure will be projected to 1.56 billion by 2025. Hypertension is a cause of 10 million deaths worldwide each year [2]. It is also the leading risk factor for cardiovascular morbidities and mortalities which is attributable to 62% of cardiovascular diseases, 49% of ischemic heart diseases, 45% of deaths caused by ischemic heart disease, and 51% of deaths caused by cerebrovascular disease [2].

Hypertension is also called “a silent killer” because it progresses to its complicated form without showing any symptoms. This nature of the disease makes the early diagnosis of the disorder difficult, before resulting in various cardiovascular and other complications unless regular and periodic screening is done. As a result of this, hypertension is usually diagnosed with complications, particularly in developing countries where there is low health-seeking behavior for a general checkup. For example, a study from Ethiopia has claimed that about 78% of hypertensive study participants, who were diagnosed during data collection, did not know their diagnosis before the survey [3].

Numerous studies indicate that hypertension is a widespread problem in Sub-Saharan Africa including Ethiopia; it has been reported to be as high as 38% [4–8]. An estimated 10 to 20 million people are affected by hypertension in this region [4].

A meta-analysis on the prevalence of HTN in Ethiopia found that it is increasing with an estimated prevalence of 19.6% [9]. In the last few years, the lifestyle of the Ethiopian population is changing due to urbanization and demographic transition [10]. As a result, risky health behaviors (smoking, alcohol consumption, sugar intake, and fatty meal commotion) are being increased and predispose the population to various noncommunicable diseases including hypertension [3, 11, 12].

Various studies done in different parts of the world have shown that the prevalence of hypertension among bank workers is very high in the general population. For instance, the prevalence of hypertension is 33.1% to 65.5% in India [13–15] and 33.1% in Iran [16]. Few studies elsewhere in Ethiopia also claimed that the prevalence of hypertension is higher among bank workers than the general population which ranges from 19.1% to 27.5% [17, 18].

Studies have also revealed various factors associated with hypertension positively and negatively. These include older age [15, 19], male sex [14, 15, 20], overweight/obesity [16, 19], physical activity [13, 19], smoking and alcohol use [14], and dietary patterns [11, 12].

Bank workers are at increased risk of hypertension than the general population because of their work nature. In Ethiopia, bank workers are supposed to spend more than 8 hours of their daytime sitting with no movement. This sedentary behavior predisposes workers to hypertension and other NCDs unless they take preventive health behaviors (engage in regular physical activity, increase fruit and vegetable intake, avoid smoking, and reduce alcohol intake, fatty meals, sugar, and salt intake). However, there is no previous study in the study area regarding the magnitude and determinants of hypertension. Therefore, the present study aimed to assess the magnitude and associated behavioral, biophysical, and sociodemographic factors of hypertension among bank workers in metropolitan cities of Amhara Regional State, Ethiopia. Indeed, the study will generate evidence having greater importance for behavior change intervention for the prevention of hypertension among this risk group of population.

## 2. Methods and Materials

### 2.1. Study Area and Period

An institution-based cross-sectional study was employed at three metropolitan cities in Amhara Regional State from September 1^st^ to October 30^th^, 2020. The region is located in the northwestern part of Ethiopia. It has 15 zones, 180 woredas, and three metropolitan cities. The metropolitan cities include Bahir Dar, Dessie, and Gondar which are located at about 552 km, 727 km, and 400 km away from Addis Ababa, the capital city of Ethiopia, respectively. In this city, there are 18 (16 private and 2 state) banks with 223 branches. On average, there are about 11 professional bank workers in each branch.

### 2.2. Population

Bank workers who were working in the metropolitan cities of Amhara Regional State of Ethiopia were the source population of this study.

### 2.3. Inclusion and Exclusion Criteria

Being a bank worker in the metropolitan cities of the Amhara Regional State of Ethiopia was an inclusion criterion, whereas bank workers who were absent during the data collection period had less than one year of experience and pregnant workers were excluded from the study.

### 2.4. Sample Size Determination and Sampling Technique

A single population proportion formula (*n* = *Z*2, *p*(1 − *p*)/*d*2) was used to compute the sample size. Since no published evidence shows the prevalence of hypertension among bank workers based on the new guideline for hypertension diagnosis in Ethiopia, 50% was used to get the maximum sample size by considering a 95% confidence interval, 5% marginal error (*d*), and 5% nonresponse rate. Hence, the minimum calculated sample size was 404. A simple random sampling technique was employed to recruit the study participants through a computer-generated number using a sampling frame that was created by collecting the list of workers at each bank from their respected main branch.

### 2.5. Data Collection Tools and Procedure

Data was collected using a pretested structured self-administered questionnaire which was adapted from the “WHO STEPS” tool developed for use in resource-limited countries for collecting data on chronic diseases and their risk factors. The questionnaire comprised sociodemographics, behavior, knowledge of CVDs, and stressful life events [21]. In addition to this, physical measurements were also used to collect data on biophysical attributes of the participants such as weight, height, and blood pressure. A digital measuring instrument (Seca 700 weight scale, Germany) was used to measure the weight of the participants. Weight measuring scales were checked and adjusted at zero levels before each measurement. Height was measured with a tape meter following the standard steps [22, 23]. BP was measured by trained personnel using a digital instrument **(Omron M4-I®)** after the subject had rested for 5 min in the sitting position having had no cigarettes, coffee, or tea. Three measurements of BP on a single visit were taken at least 3 minutes apart, and the averages of the three records were used to determine the blood pressure of the participants.

### 2.6. Study Variables

The dependent variable of this study was hypertension, whereas the independent variables of the study included sociodemographic factors (age, sex, marital status, educational status, monthly income, and religion), behavioral factors (alcohol drinking, cigarette smoking, physical activity, and daily salt, fruit, and vegetable intake), obesity, overweight, familial history of hypertension, knowledge of CVDs, and stressful life events.

### 2.7. Measurements

#### 2.7.1. BP Measurement

BP was categorized based on the recommendations of the new 2017 American College of Cardiology Guideline for the Prevention, Detection, Evaluation, and Management of High BP in Adults [24]. The classification of BP (expressed in mmHg) was done as follows: normal if systolic BP was lower than 120 and diastolic BP was lower than 80, elevated BP if systolic BP was 120–129 and diastolic BP was less than 80, stage 1 hypertension if systolic BP was 130–139 and/or diastolic BP was 80–90, and stage 2 hypertension if systolic BP was ≥140 and/or diastolic BP was ≥90 mmHg. Generally, hypertension was defined as systolic BP ≥ 130 mmHg and/or diastolic BP ≥ 80 mmHg and/or taking antihypertensive medication.

#### 2.7.2. Anthropometric Measurements

Height was measured by a tape meter (to the nearest 0.005 m) with the subject's standing position and shoes and jacket removed. Weight was measured (to the nearest 0.1 kg) by using a digital weight measuring instrument. BMI, defined as body weight in kilograms divided by the square of height in meters, was calculated as a measure of weight category. BMI (expressed in kg/m^2^) was categorized as follows: underweight, BMI < 18.5; normal weight, 18.5 ≤ BMI < 25; overweight, 25 ≤ BMI < 29.9; and obese, BMI ≥ 30 kg/m^2^.

(1) Physical activity is defined as any movement produced by skeletal muscle that requires energy expenditure. It was measured by an internationally validated questionnaire, the General Physical Activity Questionnaire (GPAQ), which is composed of 16 items. The physical activity status of the participants was categorized into high, moderate, and low physical activity as per the questionnaire guideline [25].

(2) Smoking was measured with four items measuring the patterns of smoking including smoking history, current smoking, frequency of smoking, and the number of cigarettes they used to smoke. Current smoking was defined as smoking at least once within one month before the survey and smoking history was defined as ever use of a cigarette in their lifetime [26].

(3) Alcohol was measured by using a questionnaire, the so-called AUDIT, which consisted of 10 items which are designed to assess alcohol use patterns and dependency. All items except items nine and ten have four response categories. Scores for each question range from 0 to 4, with the first response for each question (e.g., never) scoring 0, the second (e.g., less than monthly) scoring 1, the third (e.g., monthly) scoring 2, the fourth (e.g., weekly) scoring 3, and the last response (e.g., daily or almost daily) scoring 4. For questions 9 and 10, which only have three responses, the scoring is 0, 2, and 4 [27].

(4) Dietary habits *were* assessed by asking participants about their weekly salt, meat, fruit, and vegetable intake. It was measured by four items, e.g., how many times do you eat fruits in a day? How many times do you eat vegetables in a day? Participants' diet, fruit, and vegetable were classified into two categories: daily fruit or vegetable intake into “1” and less than daily fruit or vegetable intake into “0” [26].

(5) Stressful life events were measured by 12 items which assessed the participants regarding stressful life events. Each item has a Yes or No response. To the global stressful life, event measure was computed by adding each item score after all items' Yes/No responses were coded into 1 and 0, respectively. The higher score indicated the participant's exposure to an increased number of stressful life events. The internal reliability test for the items measuring the global stressful life events construct was done and its Cronbach alpha was 0.71.

(6) Knowledge of participants about CVDs was assessed by 14 items having three response categories (True, False, and I do not know). For each item, 1 point was given for “correct answer” and 0 for “wrong answer and I do not know”. Then the composite knowledge score was computed by adding each item score. The internal consistency of the items was assessed with Cronbach alpha reliability coefficient and it was 0.84. The composite knowledge score was classified into three categories based on Bloom's cut-off as follows: good if the knowledge score was between 80 and 100% (>12 points), moderate if the score was between 60 and 79% (11-12 points), and poor if the score was less than < 60% (<11 points) [28].

### 2.8. Data Quality Control

The data collection instrument was prepared in English and translated into the local language, Amharic, by two individuals who are well versed in Amharic and English languages. Backward translation was also done by English language experts to check for consistency. A pretest was done with 20 bank workers from a different site to the main study area. The necessary amendment was done based on the feedback received from participants of the pretest. Six nurses with adequate experience were recruited to collect the data. One day training was given to the data collectors on the objective of the study, the technique of data collection, the content of the questionnaire, and issue of confidentiality of the participants. Moreover, BP was measured in a quiet place and a close supervision was done by the investigators; in the meantime, reports from the data collectors were received on daily basis.

### 2.9. Data Management

Following data collection, each questionnaire was reviewed for completeness and consistency, and possible amendments were done by the investigator. Data was entered into Epi-info version 7 and transferred into STATA version 14 and then data cleaning and coding were done to make it ready for analysis.

### 2.10. Statistical Analysis

The results of the descriptive statistics were expressed as mean, standard deviation, percentage, and frequency using tables and charts. Binary logistic regression was employed to identify factors associated with hypertension. Those variables with *p*-value less than or equal to 0.2 in the bivariable analysis were a candidate for multivariable analysis. Multivariable analysis was used to control potential confounders, and to declare the signature of the association, *p*-value <0.05 was used. Moreover, the magnitude of the association between different independent variables to the dependent variable was measured using odds ratios with a 95% confidence interval. Indeed, the Hosmer–Lemeshow goodness-of-fit test was used to test the model fitness and indicated the model was well fitted to the data. Furthermore, multicollinearity between the explanatory factors was assessed with the variance inflation factor (VIF) to identify and avoid redundant variables that may affect our estimates. The VIF was in the acceptable range for all variables [1–4].

## 3. Result

A total of 368 bank employees participated in this study with a response rate of 91.1%. The mean age of the participants was 36.2 (SD = 9.9). The majority (77.5%) of the participants were males. More than half of the participants were private bank employees (Table 1).

### 3.1. Experience of Stressful Life Events

The median global stressful life events score was 1 with an interquartile range of 0 to 3. About 240 (65.2%) participants experienced at least one stressful life event in the past six months before this survey. Nearly one-fifth of the participants reported that they were suffering a serious illness, injury, or assault in the past 6 months before the survey (Table 2).

#### 3.1.1. Physical Measurements and Behavioral Factors

Nearly one-third (28%) of the participants were overweight, whereas 27 (7.3%) and 55 (16%) of the participants were underweight and obese, respectively. Regarding the feeding habit of the participants, only 32 (8.7%) and 24 (6.52%) ate vegetables and fruit at least once a day, respectively. Moreover, 280 (76.1%) and 16 (4.4%) of the participants drank alcohol and smoked a cigarette within the past one month before the survey, respectively (Table 3).

#### 3.1.2. Knowledge of Cardiovascular Diseases

Participants were asked about their knowledge of CVDs including hypertension. More than half (55.4%) of the study participants had poor knowledge of CVDs, whereas 83 (22.6%) and 81 (22%) had moderate and good knowledge of CVDs, respectively. In addition to this, only 96 (26.1%) of the participants knew their BP status before the present study.

#### 3.1.3. Prevalence of Hypertension

**Figure 1:**
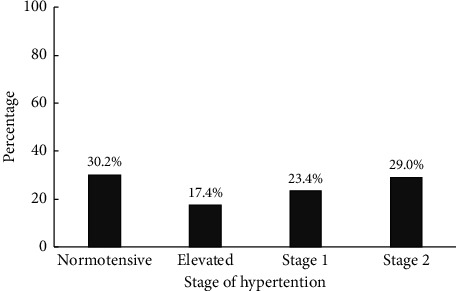
Blood pressure status of bank employees in metropolitan cities of the Amhara Regional State, Ethiopia, 2020 (*n* = 368).

The mean systolic and diastolic BP were 125.45 mmHg (SD = 14.5) and 78.95 mmHg (SD = 12.4). About one-third (32.6%) of the participants were newly diagnosed with hypertension, whereas 19.8% of the participants were known hypertensives. The overall prevalence of hypertension was 52.4% (95% CI: 47.2, 57.7), of which about 86 (44.6%) and 107 (55.4%) were stage 1 and stage 2 hypertensives as they had hypertension according to the BP classification of a guideline newly developed by the American College of Cardiology by 2017 (Figure 1).

#### 3.1.4. Factors Associated with Hypertension

Both bivariable and multivariable logistic regression analyses were done to identify factors associated with hypertension. In bivariable logistic regression analysis, each predictor variable (age, sex, educational status, marital status, religion, monthly income, BMI, alcohol intake, smoking history, fruit and vegetable intake, physical activity, meat consumption, familial history of hypertension, salt intake, social support, stressful life events, and knowledge of CVDs) was fitted to predict the outcome variable (hypertension) and all of the variables crudely associated with hypertension at a *p*-value of less than 0.2 were selected for multivariable logistic regression analysis. These included age, sex, education, monthly income, BMI, current alcohol drinking, smoking history, physical activity, stressful life events, familial hypertension, knowledge of CVDs, and daily salt, fruit, and vegetable intake.

In the multivariable logistic regression analysis, age, male sex, being overweight, being obese, engaging in moderate/high physical activity, having a familial history of hypertension, experiencing at least one stressful life event, daily fruit consumption, and knowledge of CVDs were significantly associated factors with being hypertensive.

For a year increase in the age of the participants, the odds of having hypertension was increased by 10% (AOR = 1.1, 95% CI: 1.03, 1.11). Being male doubled the odds of having hypertension (AOR = 2.5, 95% CI: 1.2, 5.1). The odds of hypertension were (AOR = 2.7, 95% CI: 1.5, 5.2) and (AOR = 5.6, 95% CI: 2.0, 11.3) times higher among overweight and obese participants than participants having BMI of <24.9 kg/m^2^, respectively. Regarding physical activity status, those participants who had moderate or high physical activity status were less likely to be hypertensive compared to participants having low physical activity status (AOR = 0.36, 95% CI: 0.2, 0.62). Moreover, the odds of having hypertension were reduced by 90% among participants who consumed fruits daily (AOR = 0.1, 95% CI: 0.04, 0.3). Participants who had experienced at least one stressful life event within six months before the survey were 1.8 times more likely to having hypertension (AOR = 1.8, 95% CI: 1.01, 3.4). The odds of having hypertension were 2.8 times higher among participants who had a family history of hypertension (AOR = 2.8, 95% CI: 1.5, 5.4). Indeed, those participating who had poor CVDs knowledge were 2.4 times more likely to have hypertension compared to those participants who had good knowledge of CVDs (AOR = 2.4, 95% CI: 1.2, 4.8) (Table 4).

## 4. Discussion

This study was conducted to assess the prevalence of hypertension and its determinants among bank workers. In the present study, the prevalence of hypertension was 52.4%, which indicated more than half of the study participants had hypertension. This finding is higher when compared to studies done elsewhere in Ethiopia [17, 18] and Nigeria [29]. However, it is lower than studies in India [14–16, 19]. The discrepancy may be due to differences in socioeconomic differences among the study participants of the present study and to those participants of studies done in other countries since hypertension is highly affected by behavioral factors. Moreover, the observed discrepancies of the present study to previous studies in Ethiopia may be attributed to the differences in a criterion used to diagnose hypertension where we used the new American College of Cardiac criteria whereas the previous studies used the joint national committee to diagnose hypertension. In addition to this, time variation could be one possible reason for the discrepancy. For instance, the aforementioned study which was done in Nigeria was conducted in 2012, almost a decade before the present study through which various sociodemographic and economic changes could happen that may increase risk health behaviors for hypertension.

Moreover, nearly half of the participants were newly diagnosed with hypertension, and about three-fourths of the total participants did not know their BP status before the present study. This is a common phenomenon claimed by several previous studies [11, 14, 17, 18, 30]. The possible reason for this might be that hypertension is usually symptomless, so people may not seek health services. It also signifies that people will end up with an advanced stage of hypertension and its fatal complications without experiencing symptoms unless periodic screening is done so that they can know their status in the early stage of the disorder and they can take prompt action to treat as well as to prevent its complications. Particularly, risk groups like bank workers could benefit more if they engage in regular screening.

Regarding the determinants of hypertension, increased age, male sex, overweight, obesity, moderate/high physical activity, familial history of hypertension, stressful life events, daily fruit consumption, and poor knowledge of CVDs were significantly associated with hypertension.

As the age of the participants increased, the probability of being hypertensive was increased. The finding is in line with studies done in Ethiopia [17], Iran [16], India [16, 19], and Hungary [30]. The observed association may be explained by aging-associated structural changes in the arteries and especially with large artery stiffness that is thought to result in an increment of BP [31].

In the present study, male participants were more likely to be hypertensive than female participants. This association was also claimed by previous studies done in Nigeria [29] and India [14, 32]. However, another study done in India has shown that there was no difference in the prevalence of hypertension across sexes [15].

Overweight or obesity is thought to be one of the risk factors that increase the risk of hypertension [33]. The present study also claimed that the prevalence of hypertension was higher among participants who were overweight and obese compared to those participants who were underweight/normal weight. This is in line with various studies done among bank workers [13, 16, 17, 19]. Although the mechanism by which obesity directly causes hypertension is under investigation, activation of the sympathetic nervous system, the amount of intra-abdominal and intravascular fat, sodium retention leading to an increase in renal reabsorption, and the renin-angiotensin system are considered to have important functions in the pathogenesis of obesity-related hypertension [33].

Moreover, the present study also claimed that participants who consumed fruits daily were less likely to have hypertension. This is similar to previous studies [11, 12]. It is also further supported by interventional studies that claim that fruit consumption is effective in lowering BP through increasing the antioxidants that are responsible for the reduction of free radicals [34, 35].

Furthermore, the likelihood of having hypertension was higher among participants who had experienced at least one stressful life event. The effect of stressful life events on hypertension is not clear. For instance, a study done among employees claimed that stressful life events are associated with hypertension [36]. On the other hand, a study done in the USA showed that there is a significant association between stressful life events and hypertension [37]. Further longitudinal studies may be beneficial to secure the effect of stressful life events on BP.

The odds of having hypertension were higher among participants who had poor knowledge of CVDs risk factors. The finding is inconsistent with studies done to investigate the effect of knowledge of CVDs on the prevention and control of hypertension [38]. This discrepancy may be due to sociodemographic and health literacy differences among the study participants. However, the observed association may be because those participants having good knowledge of CVDs may have better decision-making about their health so that they can protect themselves from being exposed to risk factors of CVDs including hypertension.

Indeed, the odds of having hypertension were higher among participants who had a familial history of hypertension compared to those participants with no familial history of hypertension. This finding is similar to studies done in India among bank workers [14, 15]. This is because hypertension has a hereditary way of transmission from parents to their offspring.

### 4.1. Limitations of the Study

It may not be possible to establish causal relationships between the predictor and outcome variable because of the cross-sectional nature of the study. In addition to this, the findings of this study may not be generalized to the general population. Measurement errors that occurred during BP, weight, and height measurements may also be another limitation of the present study. Indeed, the present study did not compare the prevalence of hypertension between workers and the general population. Thus, we strongly recommend for successive researchers to carry out a comparative/longitudinal study to precisely determine the magnitude of the risk attributed to their occupation.

### 4.2. Strength of the Study

In the presence of the aforesaid limitations, the present study addresses a vital issue among this risk group of the population for hypertension and cardiovascular diseases. The survey also tried to assess important factors of hypertension that have great importance for behavior change intervention for the prevention of hypertension among these occupationally exposed populations.

## 5. Conclusion

The prevalence of hypertension was very high (52.4%) among bank workers that signify participants are at higher risk of developing cardiovascular disorders and other multidimensional consequences of hypertension unless urgent interventions are taken. In addition to this, increased age, male sex, increased BMI, never smoke, moderate/high physical activity, familial history of hypertension, stressful life events, and daily fruit consumption were significantly associated factors with hypertension.

### 5.1. Recommendation

Dietary habits, physical exercise, and weight reduction interventions are urgently required to prevent and reduce the burden of hypertension and its consequences among this risk group of population. In addition to this, periodic screening for hypertension is highly advised for bank workers, particularly for those of older age and those who are overweight/obese. Indeed, longitudinal/comparative studies are recommended to determine the magnitude of the risk of hypertension attributable to occupational exposure.

## Figures and Tables

**Table 1:** Sociodemographic characteristics of bank employees in metropolitan cities of Amhara Regional State, Ethiopia, 2020 (*n* = 368).

Variables	Frequency	Percent
Age		
<30	121	32.9
30–39	148	40.2
≥40	99	26.9

Sex		
Male	285	77.5
Female	83	22.5

Marital status		
Married	224	60.87
Single	144	39.13

Religion		
Christian	303	82.34
Muslim	65	17.66

Education		
Diploma or first degree	280	76.09
Master's and above	88	23.91

Monthly income^C^	13816.52	200.84
Year of service		
<5	98	26.63
5–10	174	47.28
>10	96	26.09

Type of bank		
Government	249	67.66
Private	119	32.34

*Note.* C = continuous variable and for such variables, means and standard deviations are depicted in the table.

**Table 2:** Stressful life event experiences among bank employees in metropolitan cities of Amhara Regional State, Ethiopia, 2020 (*n* = 368).

Items		Frequency	Percentage
Have you suffered a serious illness, injury, or assault in the past six months?	Yes	72	19.57
	No	296	80.43
In the past six months, is there serious illness, injury or assault happened to a close relative?	Yes	80	21.74
	No	288	78.26
In the past six months, is there anyone in your family (parent, child, or spouse) who died?	Yes	48	13.04
	No	320	86.96
In the past six months, have you lost your close family friend or another relative (aunt, cousin, grandparent) because of death?	Yes	64	17.39
	No	304	82.61
Have you had a separation due to marital difficulties in the past six months	Yes	24	6.52
	No	344	93.48
Did you break off a steady relationship in the past six months?	Yes	72	19.57
	No	296	80.43
Have you had a serious problem with a close friend, neighbor, or relative in the past six months?	Yes	88	23.91
	No	280	76.1
Have you had become unemployed or you were seeking work unsuccessfully for more than one month in the past six months?	Yes	16	4.35
	No	352	95.65
Were you sacked from your job in the past six months?	Yes	16	4.35
	No	352	95.65
Have you had suffered a major financial crisis in the past six months?	Yes	96	26.1
	No	272	73.91
Have you had problems with the police and a court appearance in the past six months?	Yes	40	10.87
	No	328	89.13
Something you valued was lost or stolen in the past six months?	Yes	32	8.70
	No	336	91.30

**Table 3:** Physical measurements and behavioral characteristics of bank employees in metropolitan cities of Amhara Regional State, Ethiopia, 2020 (*n* = 368).

Items	Frequency	Percentage
BMI status		
Underweight (<18.5 kg/m^2^)	27	7.34
Normal (18.5–24.9 kg/m^2^)	183	49.73
Overweight (25–29.9 kg/m^2^)	103	27.99
Obese (≥30 kg/m^2^)	55	14.95
Mean = 24.3 kg/m^2^		
SD = 4.7		

Vegetable intake		
Never	56	15.22
1–3 per week	144	39.13
4–6 per week	136	36.96
Once a day	32	8.7

Fruit intake		
Never	24	6.5
1–3 per week	184	50
4–6 per week	136	36.96
Once a day	16	4.35
At least twice a day	8	2.17

Daily salt intake		
<one teaspoon	208	56.52
>one teaspoon	160	43.48

Ever alcohol drinking		
Yes	328	89.1
No	40	10.9

Alcohol intake during the last month		
Yes	280	76.1
No	88	23.9

Ever cigarette smoking		
Yes	56	15.2
No	312	84.8

Smoking in the last month		
Yes	16	4.4
No	352	95.6

Physical activity		
Low	185	50.3
Moderate	165	44.8
High	18	4.9

Familial history of hypertension		
Yes	80	21.7
No	288	28.3

**Table 4:** Factors associated hypertension among bank employees in metropolitan cities of Amhara Regional State, Ethiopia, 2020 (*n* = 368).

Variables	Hypertension, *n* (%)		COR (95% CI)	AOR (95% CI)
	No	Yes		
Age^C^, mean (SD)	32.8 (7.95)	39.9 (10.5)	1.1 (1.05, 1.11)	1.1 (1.03, 1.11)^*∗∗*^
Sex				
Male	129	156	1.5 (0.9, 2.5)	2.5 (1.2, 5.1)^*∗*^
Female	46	37	1	1

Education				
Diploma/BA	126	154	1.5 (0.9, 2.5)	1.4 (0.7, 2.6)
MA	49	39	1	1

Monthly income^C^	1355.5 (3956.9)	14101 (3726.3)	1 (0.9, 1.01)	1 (0.9, 1.01)
BMI status				
Underweight/normal	131	79	1	1
Overweight	31	72	3.8 (2.3, 6.3)	2.7 (1.5, 5.2)^*∗∗*^
Obese	13	42	5.3 (2.7, 10.5)	5.6 (2.0, 11.3)^*∗∗*^

Current alcohol drinking				
Yes	126	154	1	1
No	49	39	0.7 (0.4, 1.1)	0.9 (0.4, 1.8)

Smoking history				
Yes	21	35	1	1
No	154	158	0.6 (0.3, 1.1)	1.2 (0.5, 2.7)

Physical activity				
Low	56	129	1	1
Moderate/high	119	64	0.23 (0.2, 0.4)	0.36 (0.2, 0.62)^*∗∗*^

Daily salt intake				
<1 tea spoon	111	97	1	1
≥1 tea spoon	64	96	1.4 (0.9, 2.2)	1.7 (0.9, 2.9)

Daily fruit intake				
Yes	17	7	0.4 (0.1,0.9)	0.1 (0.04, 0.3)^*∗∗*^
No	158	186	1	1

Daily vegetable intake				
Yes	19	13	0.6 (0.3, 0.1.2)	0.9 (0.4, 2.6)
No	156	180	1	1

Stressful life event				
No	74	54		
At least one LTE	101	139	1.9 (1.2, 2.9)	1.8 (1.01, 3.4)^*∗*^

Familial hypertension		1	1	
Present	22	58	3 (1.7, 5.1)	2.8 (1.5, 5.4)^*∗∗*^
Absent	153	135	1	1

Knowledge of CVDs				
Poor	79	125	2.4 (1.4, 4.1)	2.4 (1.2, 4.8)^*∗*^
Moderate	47	36	1.2 (0.6, 2.1)	1.6 (0.8, 3.8)
Good	49	32	1	1

**Key:** C = continuous variable, ^*∗*^*P* < 0.05, ^*∗∗*^*P* < 0.01, LTE = life threatening events, BMI = body mass index, COR = crude odds ratio, AOR = adjusted odds ratio, and SD = standard deviation. *Note.* The Hosmer–Lemeshow goodness-of-fit test was chi2(8) = 5.2 Prob > chi2 = 0.73, which indicated the model was well fitted to the data.

## Data Availability

The data that support the findings of this study are available from the corresponding author (KS) upon reasonable request.

## Abbreviations

AOR: Adjusted odds ratio
BMI: Body mass index
BP: Blood pressure
CI: Confidence interval
COR: Crude odds ratio
CVDs: Cardiovascular diseases
mmHg: Millimeter mercury
kg: Kilogram.

## References

[1] WHO (2019). *Hypertension*.

[2] Global W. HO. (2013). *Brief on Hypertension: Silent Killer, Global Public Health Crisis: WHO Day 2013*.

[3] Haye T. B., Tolera Agama B. (2020). Prevalence of hypertension and associated factors among the outpatient department in akaki kality subcity health centers, Addis Ababa, Ethiopia. *International Journal of Hypertension*.

[4] Opie L. H., Seedat Y. K. (2005). Hypertension in sub-saharan african populations. *Circulation*.

[5] Addo J., Smeeth L., Leon D. A. (2007). Hypertension in sub-saharan Africa. *Hypertension*.

[6] Yoruk A., Boulos P. K., Bisognano J. D. (2018). The state of hypertension in sub-saharan Africa: review and commentary. *American Journal of Hypertension*.

[7] Guwatudde D., Nankya-Mutyoba J., Kalyesubula R (2015). The burden of hypertension in sub-saharan Africa: a four-country cross sectional study. *BMC Public Health*.

[8] Ataklte F., Erqou S., Kaptoge S., Taye B., Echouffo-Tcheugui J. B., Kengne A. P. (2015). Burden of undiagnosed hypertension in sub-saharan Africa. *Hypertension*.

[9] Kibret K. T., Mesfin Y. M. (2015). Prevalence of hypertension in Ethiopia: a systematic meta-analysis. *Public health Reviews*.

[10] Strengthening F. M. O. (2011). Chronic disease services in Ethiopia: lessons learned from HIV/AIDS program implementation. *National Conference & Exhibition*.

[11] Kebede B., Ayele G., Haftu D., Gebremichael G. (2020). The prevalence and associated factors of hypertension among adults in southern Ethiopia. *International Journal of Chronic Diseases*.

[12] Chuka A., Gutema B. T., Ayele G., Megersa N. D., Melketsedik Z. A., Zewdie T. H. (2020). Prevalence of hypertension and associated factors among adult residents in arba minch health and demographic surveillance site, southern Ethiopia. *Plos One*.

[13] Savani N., Chauhan R., Chudasama R. (2020). A study to assess the prevalence and risk factors of hypertension among the bank employees of rajkot city, Gujarat, India. *National Journal of Community Medicine*.

[14] Brahmankar T. R., Prabhu P. M. (2017). Prevalence and risk factors of hypertension among the bank employees of Western Maharashtra-a cross sectional study. *International Journal Of Community Medicine And Public Health*.

[15] Ismail I., Kulkarni A., Kamble S., Rekha R., Amruth M., Borker S. (2013). Prevalence of hypertension and its risk factors among bank employees of Sullia Taluk, Karnataka. *Sahel Medical Journal*.

[16] Montazerifar F., Karajibani M., Abbasi M., Bolouri A. (2019). Prevalence of obesity and hypertension and related factors among bank employees in zahedan, 2017. *International Journal of Epidemiologic Research*.

[17] Gt Z. (2020). Prevalence of hypertension and associated factors among bank workers in harar town , eastern Ethiopia 2018. *Journal of Community Medicine & Public Health*.

[18] Fikadu G., Lemma S. (2016). Socioeconomic status and hypertension among teachers and bankers in addis Ababa, Ethiopia. *International Journal of hypertension*.

[19] Maroof K. A., Parashar P., Bansal R., Ahmad S. (2007). A study on hypertension among the bank employees of Meerut district of Uttar Pradesh. *Indian Journal of Public health*.

[20] Tayem Y. I., Yaseen N. A., Khader W. T., Abu Rajab L. O., Ramahi A. B., Saleh M. H. (2012). Prevalence and risk factors of obesity and hypertension among students at a central university in the West bank. *The Libyan Journal of Medicine*.

[21] The WHO STEP wise approach to noncommunicable disease risk factor surveillance [Internet]. Available from: https://www.who.int/ncds/surveillance/steps/STEPS_Manual.pdf?ua=1

[22] Tesfaye F., Byass P., Wall S. (2009). Population based prevalence of high blood pressure among adults in Addis Ababa: uncovering a silent epidemic. *BMC Cardiovascular Disorders*.

[23] Gudina E., Assegid S., Michael Y. (2013). Prevalence of hypertension and its risk factors in southwest Ethiopia: a hospital-based cross-sectional survey. *Integrated Blood Pressure Control*.

[24] Whelton P. K., Carey R. M., Aronow W. S (2017). *ACC/AHA/AAPA/ABC/ACPM/AGS/APhA/ASH/ASPC/NMA/PCNA Guideline for the Prevention, Detection, Evaluation, and Management of High Blood Pressure in Adults: Executive Summary: A Report of the American College of Cardiology*.

[25] WHO (2012). *Global Physical Activity Questionnaire (GPAQ) Analysis Guide*.

[26] Negesa L. B., Magarey J., Rasmussen P., Hendriks J. M. L. (2020). Patients’ knowledge on cardiovascular risk factors and associated lifestyle behaviour in Ethiopia in 2018: a cross-sectional study. *Plos one*.

[27] Lawford B. R., Barnes M., Connor J. P., Heslop K., Nyst P., Young R. M. (2012). Alcohol use disorders identification test (AUDIT) scores are elevated in antipsychotic-induced hyperprolactinaemia. *Journal of Psychopharmacology*.

[28] Akalu Y., Ayelign B., Molla M. D. (2020). Knowledge, attitude and practice towards covid-19 among chronic disease patients at addis zemen hospital, Northwest Ethiopia. *Infection and Drug Resistance*.

[29] Diwe K. C., Enwere O. O., Uwakwe K. A., Duru C. B., Chineke H. N. (2015). Prevalence and awareness of hypertension and associated risk factors among bank workers in Owerri, Nigeria. *International Journal of Medicine and Biomedical Research*.

[30] Sonkodi B., Sonkodi S., Steiner S. (2012). High prevalence of prehypertension and hypertension in a working population in Hungary. *American Journal of Hypertension*.

[31] Pinto E. (2007). Blood pressure and ageing. *Postgraduate Medical Journal*.

[32] Abariga S. A., Khachan H., Al Kibria G. M. (2020). Prevalence and determinants of hypertension in India based on the 2017 ACC/AHA guideline: evidence from the India national family health survey. *American Journal of hypertension*.

[33] Jiang S.-Z., Lu W., Zong X.-F., Ruan H.-Y., Liu Y. (2016). Obesity and hypertension. *Experimental and Therapeutic Medicine*.

[34] England T. N. (2015). Numb Er 16 a clinical trial of the effects of dietary patterns on blood pressure.

[35] John J., Ziebland S., Yudkin P., Roe L., Neil H. (2002). Effects of fruit and vegetable consumption on plasma antioxidant concentrations and blood pressure: a randomised controlled trial. *The Lancet*.

[36] Tennant C. (2001). Life stress and hypertension. *European Journal of Cardiovascular Prevention & Rehabilitation*.

[37] Sparrenberger F., Fuchs S. C., Moreira L. B., Fuchs F. D. (2008). Stressful life events and current psychological distress are associated with self-reported hypertension but not with true hypertension: results from a cross-sectional population-based study. *BMC Public Health*.

[38] Burger A., Pretorius R., Fourie C. M. T., Schutte A. E. (2016). The relationship between cardiovascular risk factors and knowledge of cardiovascular disease in African men in the North-West province. *Health SA Gesondheid*.

